# Management and Outcomes of Traumatic Liver Injury: A Retrospective Analysis from a Tertiary Care Center Experience

**DOI:** 10.3390/healthcare12020131

**Published:** 2024-01-06

**Authors:** Tariq Alanezi, Abdulmajeed Altoijry, Aued Alanazi, Ziyad Aljofan, Talal Altuwaijri, Kaisor Iqbal, Sultan AlSheikh, Nouran Molla, Mansour Altuwaijri, Abdullah Aloraini, Fawaz Altuwaijri, Mohammed Yousef Aldossary

**Affiliations:** 1 College of Medicine, King Saud University, Riyadh 11322, Saudi Arabia438102566@student.ksu.edu.sa (Z.A.); 2 Division of Vascular Surgery, Department of Surgery, College of Medicine, King Saud University, Riyadh 11322, Saudi Arabia; taltuwaijri@ksu.edu.sa (T.A.); kiqbal@ksu.edu.sa (K.I.); sualsheikh@ksu.edu.sa (S.A.); myaldossary@moh.gov.sa (M.Y.A.); 3 Department of Radiology and Medical Imaging, College of Medicine, King Saud University, Riyadh 11322, Saudi Arabia; nmolla@ksu.edu.sa; 4 Division of Gastroenterology, Department of Medicine, College of Medicine, King Saud University, Riyadh 11322, Saudi Arabia; maltuwaijri@ksu.edu.sa; 5 Division of General Surgery, Department of Surgery, College of Medicine, King Saud University, Riyadh 11322, Saudi Arabia; abdaloraini@ksu.edu.sa; 6 Department of Emergency Medicine, College of Medicine, King Saud University, Riyadh 11322, Saudi Arabia; faltuwaijri@ksu.edu.sa; 7 Division of Vascular Surgery, Department of Surgery, Dammam Medical Complex, Dammam 32245, Saudi Arabia

**Keywords:** trauma, liver injury, grading, nonoperative management, hemodynamic stability

## Abstract

Background: although liver injuries are one of the most critical complications of abdominal trauma, choosing when to operate on these injuries is challenging for surgeons worldwide. Methods: We conducted a retrospective analysis of liver injury cases at our institution from 2016 to 2022 to describe the operative and nonoperative management (NOM) outcomes in patients with traumatic liver injuries. Baseline patient characteristics, liver injury details, treatments, and outcomes were analyzed. Results: Data from 45 patients (male, 77.8%) were analyzed. The mean age was 29.3 years. Blunt trauma was the most common injury mechanism (86.7%), whereas penetrating injuries were 8.9% of cases. Conservative management was associated with 18.9% of complications. The overall complication rate was 26.7%; delirium and sepsis were the most common (13.3%), followed by acute renal failure (4.4%), pneumonia, biliary leaks, and meningitis/seizures. Conclusions: Notwithstanding its limitations, this retrospective analysis demonstrated that NOM can serve as a safe and effective strategy for hemodynamically stable patients with liver trauma, irrespective of the patient’s injury grade. Nevertheless, careful patient selection and monitoring are crucial. Further investigations are necessary to thoroughly evaluate the management of traumatic liver injuries, particularly in the context of multiorgan injuries.

## 1. Introduction

Trauma is a substantial global public health issue and a leading cause of death, hospital admissions, and long-term disability during the first four decades of life. Additionally, it ranks among the primary causes of mortality across all age groups [1,2]. Specifically, abdominal trauma ranks as the second leading cause of death among polytrauma patients, trailing head trauma as the primary cause and proceeding thoracic injuries [1]. Furthermore, the abdominal region is the third most commonly injured body area, with approximately 25% of all abdominal trauma cases eventually requiring surgical exploration [3].

Abdominal trauma is generally divided into two main categories: blunt or penetrating. The ramifications of blunt trauma can extend to any organ, and its consequences may not always be clinically apparent, necessitating a careful and thorough investigation [2]. The primary causes of blunt abdominal injuries often involve incidents like motor vehicle accidents, falls from elevated heights, and physical attacks, among other possibilities. On the other hand, penetrating trauma is typically attributed to gunshot wounds and stabbings, which are the most frequent causes [3]. The prevalence of liver injury in patients with blunt trauma is up to 8% [4]. The liver is considered one of the most commonly injured solid abdominal organs owing to its increased size, high vascularity, unique location, weak parenchyma, and fragile capsule [5].

Due to the high risk of blood loss in patients with abdominal trauma, ultrasound is also frequently used for assessing fluid status via inferior vena cava evaluation, both in subcostal and transhepatic view [6,7]. In acute life-threatening conditions, trauma can be assessed immediately using extended-focused sonography assessment in trauma (E-FAST). E-FAST has been widely accepted and utilized in assessing trauma cases by emergency physicians and trauma surgeons alike [8]. However, in assessing the extent or grade of hepatic injuries, computed tomography (CT) is the mainstay modality, as CT findings may include lacerations, contusions, parenchymal hematoma, devascularization, subcapsular hematoma, hemoperitoneum, active bleeding, pseudoaneurysm of the hepatic artery, bile leak, and periportal edema [9].

The management of liver injuries is complicated, as it considers many essential variables, such as the patient’s hemodynamic stability and serum pH. The management can be divided into operative management (OM) and nonoperative management (NOM); nonoperative conservative management is the mainstay of treatment for hemodynamically stable healthy individuals [10]. On the other hand, OM of traumatic liver injuries should be performed when NOM fails and is considered the first-line treatment for hemodynamically unstable patients [11]. Additionally, the presence of other organ injuries and perforating liver injuries also necessitate surgical intervention. The main goal of surgical intervention is to control bleeding, prevent bile leakage, and remove necrotized tissue [11,12]. However, surgical intervention is time-bound, and decisions must be made promptly, as any delay or hesitancy may increase mortality risk [12].

A different approach to dealing with hepatic injuries includes hepatic artery embolization, especially if contrast extravasation is noted on a CT scan, regardless of the patient’s hemodynamic status [13]. While OM is commonly regarded as the preferred treatment in instances of hemodynamic instability, it is noteworthy to observe that the overall mortality rate tends to be significantly higher among patients subjected to OM compared to those undergoing NOM [11]. The decision to operate on traumatic liver injuries, especially blunt trauma, is challenging for surgeons worldwide, as a list of decision criteria and/or risk factors for NOM complications and transition to OM is not widely known. Therefore, in this study, we aimed to present the mechanism, type, and extent of injuries in patients with liver trauma and describe outcomes in the operative and nonoperative management groups.

## 2. Methods

### 2.1. Study Design

This study retrospectively examined data from 45 patients with traumatic liver injuries who presented to our hospital from January 2016 to December 2022.

The current practice of our institute regarding the management of liver trauma aligns with the World Society of Emergency Surgery (WSES) guidelines, where patients who are hemodynamically stable at presentation are treated with NOM, irrespective of their injury grade. On the other hand, hemodynamically unstable patients are generally treated with OM. The conservative management entails the ongoing monitoring of liver function tests (LFTs), blood tests, and hemoglobin levels. This involves diligent observation of patients in either the ward or the intensive care unit (ICU), as well as the administration of blood products and intravenous fluids as necessary.

The inclusion criterion was traumatic liver injury presentation on the CT scan. Pregnant patients and those with negative CT scans for liver injury were excluded.

### 2.2. Data Mining and Processing

The process of data collection involved the extraction of pertinent variables from patients’ medical records, recorded and organized through a computerized data sheet for thorough analysis and examination. Therefore, we aimed to describe baseline patient characteristics, liver injury details, treatments, and outcomes.

Variables included the following items:

- Demographic data;
- Mechanisms of injury: blunt trauma, penetrating, and iatrogenic;
- Type of injury: laceration, contusion/hematoma, and hemoperitoneum;
- Grades of injury on CT using the American Association for the Surgery of Trauma (AAST) liver injury scale;
- Associated extra-abdominal injuries;
- Hemodynamic statuses at presentation;
- Extended Focused Assessment with Sonography in Trauma (E-FAST);
- Interventional radiology procedures: angioembolization; percutaneous transhepatic biliary drainage (PTD);
- Complications;
- Length of ward, hospital, and ICU stay;
- Laboratory values at first admission, including alanine aminotransferase (ALT) and aspartate aminotransferase (AST) levels;
- Glasgow coma scale (GCS) score.

Moreover, for descriptive aims, management was divided into conservative, conservative-to-laparotomy, and laparotomy approaches. Although the conservative strategy did not provide surgical intervention, individuals who underwent minimally invasive procedures such as hepatic artery embolization and percutaneous transhepatic drainage [PTD] were included. Patients who underwent laparotomy within 12 h of arrival were assigned to the laparotomy group. Those initially offered conservative management but who later required surgery beyond 12 h of arrival formed the conservative-to-laparotomy group.

Rebleeding was defined as a significant decrease from the baseline hemoglobin, with or without hemodynamic changes, given that it was not a complication of the associated injuries or their management.

### 2.3. Statistical Analysis

Descriptive statistics such as numbers (*n*), percentages (%), means, and medians were used to summarize various aspects of the dataset. Mean ± SD (standard deviation) was employed for continuous variables. Median (min–max) was used for variables with non-normal distributions. Data were analyzed using the Statistical Package for Social Sciences version 26 (IBM Corp, Armonk, NY, USA).

## 3. Results

The mean age of the patients was 29.3 [standard deviation (SD), 15.5], with most patients being males (77.8%). Blunt trauma was the most common injury mechanism (86.7%), whereas penetrating injuries accounted for 8.9% of cases. The most dominant injury type was laceration, recorded in approximately (97.8%) of the patients, followed by contusion/hematoma in 51.1%. Regarding liver injury severity, the most common grade in our patient population was 3, accounting for 37.8% of the patients (Table 1).

Most patients suffered associated injuries (*n* = 41, 91.1%), with thoracic injuries being the most common (77.8%) (Table 2).

**Figure 1:**
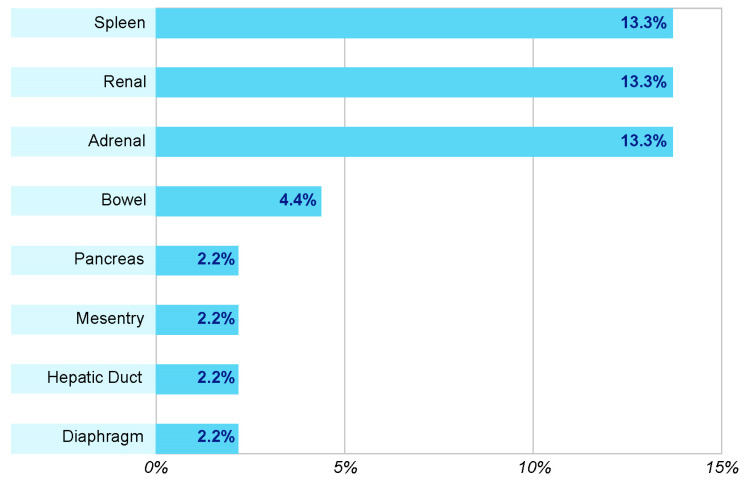
Associated abdominal organ injuries experienced by the patients.

Figure 1 shows the associated abdominal organ injuries experienced by patients. The most commonly reported intra-abdominal injuries were those to the spleen, kidney, and adrenal glands (13.3%).

### Treatment and Outcomes of Patients

Most patients (82.2%) underwent conservative management and did not require surgical intervention. Among patients managed conservatively, three underwent interventional radiological procedures, including hepatic artery angioembolization in two patients and conservative management with drainage through PTD insertion in one patient. Of the patients who were managed surgically, laparotomy was used in 13.3% (*n* = 6), and 4.4% (*n* = 2) underwent conservative-to-laparotomy management.

At presentation, 31 (68.9%) and 14 (31.1%) patients were hemodynamically stable and unstable, respectively. The overall mortality rate was 2.2% (*n* = 1). Multiple parameters were measured immediately upon admission to the emergency department. The median rebleeding rate was 1 (average: 1–5 times). Twenty-five patients required transfusion with blood products. Regarding the hospitalization course, the median length of the total hospital stay was 11 days (range: 1–316), whereas the median length of ICU stay for all patients was 4 days (range: 1–54). An expected difference was observed in the length of ICU and ward stay, as the median of the latter was 7 days (range: 1–262).

Surgically managed individuals either directly underwent laparotomy (*n* = 6, 13.3%) or underwent conservative management first and then required laparotomy, with at least a 12-hour gap between admission and surgery (*n* = 2, 4.4%). Among those who directly underwent laparotomy (*n* = 6), two had grade 4, two had grade 3, and the remaining two had grade 1 and 2 injuries, respectively. Four of these patients were hemodynamically unstable at presentation, and one presented with a penetrating gunshot wound associated with a massive diaphragmatic injury, necessitating prompt surgical repair. The remaining patient had a history of a second-story window fall that resulted in high-grade liver injury and signs of retroperitoneal hemorrhage (Table 3).

The main characteristics of the OM and NOM groups are reported in Table 4.

The variables linked to hemodynamic status and outcome based on the patient’s baseline characteristics, complications, ICU, and ward stays, as well as GCS score, are presented in Table 5.

The rate of complications in this study was 26.7%, with sepsis and delirium being the most commonly reported, occurring in 13.3% of the patients. This was followed by acute renal failure, pneumonia, and seizures, each occurring in 4.4% of patients. Cardiac arrest, meningitis, splenic infarction, disseminated intravascular coagulation, and acute respiratory distress syndrome have been reported in some patients. The most common complication was delirium, which is attributable to multiple underlying factors, including head trauma, a history of substance abuse and withdrawal, and sepsis.

Moreover, biliary leaks were less frequently reported, being documented in only four patients. Two of them underwent endoscopic retrograde cholangiopancreatography (ERCP). In the first case, the ERCP of the patient showed a proximal common bile duct leak; as a result, common bile duct and pancreatic duct stenting was performed. In the second case, the patient underwent endoscopic stent insertion for a gastric leak, as well as ERCP for the biliary leak that was present in the patient’s drain. In the two remaining patients, one had developed a complete cut-off of the common hepatic duct, which resulted in a subhepatic collection that required drainage via PTD, while the other had an intra-operatively apparent bile leak, for which an abdominal washout and repair were performed.

## 4. Discussion

Liver injuries are the most common cause of death in trauma cases because of the large adjacent vascular structures. They are the second most frequent solid organ injuries after blunt abdominal trauma [14]. In the present study, we assessed the management and outcomes of liver injury cases in a tertiary care center.

At our center, we rely on the AAST Liver Injury Scale, 2018 version, which categorizes liver injuries into five grades based on the 2018 version of the AAST Liver Injury Scale, depending on the CT findings and operative and pathological criteria [15]. Grade 3 was the most frequently observed injury. More recently, the World Society of Emergency Surgery (WSES) 2020 classified liver injuries into four main categories, including minor (WSES grade 1), moderate (WSES grade 2), and severe (WSES grades 3 and 4), considering the AAST liver injury scale score and hemodynamic status of the patients [11].

The incidence of liver trauma mechanisms varies between studies. However, a large quarter-century study of liver trauma uncovered that blunt abdominal trauma was more common than penetrating injuries [16]. Additionally, a comprehensive study of 749 patients revealed blunt abdominal trauma as the predominant injury type, accounting for 94% of cases, with the liver being the most frequently injured abdominal organ [17]. This is consistent with our study, which revealed that blunt abdominal trauma was the most common etiology of liver injury (86.7%).

Following blunt abdominal trauma, penetrating injuries constituted 8.9% of the cases, and iatrogenic injuries accounted for 4.4% of the total cases in our study. These results were similar to Petrowsky et al.’s findings, who found in their 25-year study that included 468 patients that blunt trauma was the most common cause of liver injury (84%), whereas penetrating injuries accounted for only 16% of the cases [16].

In this study, the demographic characteristics of the cohort experiencing traumatic liver injury revealed a predominant representation of young adults, predominantly males. These findings align closely with the research conducted by Hommes et al. [18], where a similar pattern was observed. Specifically, they reported a mean age of 29 years among their study participants, and a substantial majority of 72% were males.

Additionally, this study demonstrated the majority of our patients had associated injuries, with thoracic being the most common, followed by orthopedic and head injuries. Other studies have reported similar findings, with one study showing thoracic injuries as the most prevalent associated injury, followed by extremity and head trauma [19]. Another study focusing on surgically treated patients with liver injury observed that musculoskeletal injuries are the most frequent, followed closely by thoracic injuries [20]. These consistent findings underscore the multifaceted nature of traumatic incidents, emphasizing the importance of considering and addressing concomitant injuries in the overall management and care of patients with traumatic liver injuries.

NOM is generally considered the standard of care for blunt liver trauma, with >95% of these injuries being non-surgically managed at a success rate of 80–100% [18]. However, the major determinants of the NOM approach are hemodynamic stability and absence of peritoneal irritation or other internal injuries requiring surgery, irrespective of the initial injury grade [11,19,21]. Implementing these strict inclusion criteria for NOM has yielded remarkable improvements in patient outcomes. A study evaluating 97 patients who underwent NOM for blunt abdominal trauma demonstrated notable findings, as none succumbed to abdominal injuries or required conversion to OM [22]. Our findings were similar, with 82.2% of the patients managed conservatively and none experiencing cardiac arrest. Significantly, even at higher liver injury grades such as 3, 4, and 5, these patients were nonoperatively managed, and none died. These results were similar to those of Sinha et al., with 71.2% of their patients undergoing NOM at a success rate of 90% [23]. Moreover, a prospective Saudi study by Ghnnam et al. [24] that evaluated liver trauma patients over four years revealed that conservative management had a success rate of 100%. Additionally, Yildirim et al. [19] retrospectively analyzed NOM in 104 patients with liver injuries. The study showed that the NOM was successful in 94 patients, whereas surgical management was performed in 10 patients in whom NOM failed. Moreover, another study revealed that among 181 traumatic liver injury cases, 96.7% of patients successfully underwent NOM [25]. Taken together, these findings highlight the substantial success potential of the NOM in a considerable number of patients, particularly when precisely implemented within centers that adhere to a stringent NOM inclusion protocol.

The major concern for conservative management is the possibility of missing other less clinically apparent injuries that are unclear on CT imaging [26]. Modern methods of managing hepatic injury utilize interventional radiologists, as they are becoming integral in NOM. An increasing shift towards angioembolization has been observed in patients with contrast extravasation on CT scans and hemodynamic stability [27]. In our study, interventional radiology was crucial, as angioembolization was performed in two patients to control active bleeding following their decreased hemoglobin levels.

Despite the growing trend towards using NOM for liver trauma, OM remains the cornerstone of treating hemodynamically unstable patients with hepatic injuries, irrespective of their initial injury grade [10,11]. Interestingly, we observed no significant difference in the liver injury grade between patients who underwent OM and NOM. Notably, among patients managed with laparotomy or conservative-to-laparotomy methods, grade 2 injury was the most common. Despite the low injury grade, half of the patients in the OM cohort presented to the emergency department with hemodynamic instability. Some had associated organ injuries, and most had positive E-FAST findings, supporting the decision to use OM despite their low injury grade.

Similarly, another study focusing on the management of blunt abdominal trauma reported no significant disparity in the liver injury grade between patients managed operatively and those managed nonoperatively [22]. These findings highlight the importance of considering hemodynamic status and the presence of other internal organ injuries rather than solely focusing on the injury grade when determining the need for operative management.

However, it should be noted that surgical treatment for liver injuries is associated with higher morbidity and mortality [10]. In our study, eight patients required surgical interventions, and NOM had failed in two, who were subsequently taken to the operating theater, while the remaining patients were managed directly via laparotomy. Of these patients, blunt trauma was the most common mechanism of injury, and E-FAST was positive in 85.7% of the total cases that required surgical intervention. This rate of OM failure was similarly reported in a study by Jyothiprakasan et al. [28], which included 70 patients with liver trauma, 11 of whom required immediate surgical management, and five had NOM failure.

Multiple prognostic factors are important in liver trauma. Nishida et al. [29] revealed that the GCS, postoperative blood urea nitrogen, number of associated injured organs, preoperative ALT levels, and systolic blood pressure readings were significant prognostic factors in a multivariate analysis. Similarly, we observed lower GCS scores in hemodynamically unstable patients, as well as higher blood transfusion requirements. Regarding the duration of a hospital stay, we noted that the median days of ICU and ward stays and total lengths of hospital stays were longer in those requiring OM, both for immediate laparotomy and NOM failure that subsequently required laparotomy. This has been demonstrated in several studies, with one Chinese study reporting that the median hospitalization duration of patients who underwent NOM and those requiring urgent laparotomy was 25 and 27 days, respectively [30]. Additionally, the length of stay among patients stratified according to their hemodynamic status, the median stays in ICU and wards, and overall length of hospital stay are longer in those with unstable hemodynamics at presentation. These results were also found by Afifi et al. [31], who reported that patients who underwent OM had a longer ICU stay and total length of hospital stay than those only undergoing NOM.

In addition to long hospital stays, complications following hepatic trauma, from simple fever to sepsis and acute respiratory distress syndrome, were observed. This broad range of complications seems to be more frequent in hemodynamically unstable patients and those who underwent OM. Delirium was the most prevalent complication. Notably, patients with changes in their level of consciousness were comprehensively assessed, and the Mini-Mental State Examination and psychiatric consultation were performed to evaluate and diagnose delirium. The intricacies underlying delirium following trauma are complex and involve multiple factors. A study of delirium development following trauma identified abdominal surgery as the most significant risk factor. Additional risk factors for developing delirium include advanced age, male sex, lower hemoglobin levels, and a prolonged ICU stay [32]. However, the aforementioned study included all abdominal trauma cases, excluding those with concomitant head injuries. In contrast, our study included a significant proportion of patients with concomitant head injuries and individuals with substance abuse histories who experienced withdrawal upon admission. Moreover, a notable portion (13.3%) of our patients developed sepsis, potentially exacerbating their overall condition.

Furthermore, biliary leaks were infrequently encountered in our cohort of patients, with only four cases identified. These patients were managed with either ERCP or PTD insertion to help alleviate any subhepatic collections. One of these patients’ biliary leaks was drained using PTD to alleviate a subhepatic collection, and in the remaining case, the biliary leak was intra-operatively apparent, for which an abdominal washout and repair were performed. A 10-year retrospective analysis of 398 patients with liver injury revealed that patients who developed biliary leaks were similarly managed and treated with ERCP and PTD [33]. Additionally, it is worth noting that liver-related complications of hepatic injury are less common among patients treated conservatively than those treated with OM [34]. Mortality concerning liver injuries is divided into two types: early death—usually related to hemorrhage or significant vascular compromise—and late death. The mortality rate differs (1–40%) based on the mechanism of injury and associated injuries. Late death can result from sepsis, closed head injury, or multiple organ dysfunction syndrome [35]. Among the 45 patients enrolled in our study, only one died. The exact cause of death could not be directly determined; however, acknowledging the multiple concomitant injuries that likely contributed to the patient’s death is essential. These included diffuse axonal injury, multiple intracranial hemorrhages, and descending thoracic aortic transection—all potential factors in the patient’s unfortunate outcome.

### Study Limitations

A key limitation of our study was its relatively small sample size. Moreover, this study was exclusively carried out at a single tertiary care center, where the data retrieved and analyzed were retrospective. The small sample size and the retrospective observational study design did not allow us to treat the two groups (OM and NOM) homogeneously. Moreover, the varying severity of the pathology would have introduced a significant selection bias. Therefore, we have described the clinical–demographic phenomena without making a comparison between the groups. This approach allows for an overview of the phenomenon through the evaluation of multiple variables. Notably, the dataset could be expanded and utilized for predictive analyses by using different data-driven prediction models [36].

## 5. Conclusions

Managing liver trauma is a critical and challenging aspect of trauma care. A successful outcome requires a comprehensive and coordinated multidisciplinary approach that involves experts from various fields, including trauma surgeons, critical care specialists, interventional radiologists, anesthesiologists, and others. Assessing the optimal course of action and determining whether to proceed with surgery can be challenging. Despite limitations, this retrospective analysis revealed that NOM can be a safe and effective strategy for hemodynamically stable patients with liver trauma and implemented even in patients with high injury grades. However, careful patient selection and monitoring are critical, and surgical intervention remains an important option for those who do not meet NOM criteria or experience complications. Encouraging further multicenter studies to comprehensively assess the management of traumatic liver injuries within the context of multiorgan injury will further advance our understanding of optimal treatment strategies and outcomes. In a field where obtaining validation and evidence is crucial, this study could serve as a guide for further investigations.

## Figures and Tables

**Table 1:** Baseline characteristics of the patients (*n* = 45).

Study Variables	*n* (%)
Age in years (mean ± SD)	29.3 ± 15.5
Gender	
Male	35 (77.8%)
Female	10 (22.2%)
Mechanism of injury	
Blunt trauma	39 (86.7%)
Penetrating	4 (8.9%)
Iatrogenic	2 (4.4%)
Type of injury ^†^	
Laceration	44 (97.8%)
Contusion/hematoma	23 (51.1%)
Hemoperitoneum	13 (28.9%)
Grade	
Grade 1	5 (11.1%)
Grade 2	13 (28.9%)
Grade 3	17 (37.8%)
Grade 4	9 (20%)
Grade 5	1 (2.2%)

*n*, Numbers; SD, standard deviation. ^†^ Some patients have multiple types of injury.

**Table 2:** Associated abdominal and extra-abdominal injuries (*n* = 41).

Study Variables	*n* (%)
Associated extra-abdominal injuries ^†^	
Thoracic injury	35 (77.8%)
Orthopedic injury	23 (51.1%)
Head injury	22 (48.9%)
Abdominal injury	18 (40%)
Vascular injury	6 (13.3%)
Ophthalmic injury	5 (11.1%)

*n*, Numbers; SD, standard deviation. ^†^ Some patients have multiple associated injuries.

**Table 3:** Treatments and outcomes of the patients (*n* = 45).

Variables	*n* (%)
Treatment performed	
Conservative	37 (82.2%)
Laparotomy	6 (13.3%)
Conservative to laparotomy	2 (4.4%)
Interventional radiology procedures	
Angioembolization	2 (4.4%)
PTD	1 (2.2%)
Hemodynamic stability	
Unstable	14 (31.1%)
Stable	31 (68.9%)
E-FAST	
Not performed	3 (6.7%)
Positive	16 (35.6%)
Negative	26 (57.8%)
Complications	12 (26.7%)
Specific complication	
Delirium	6 (13.3%)
Sepsis	6 (13.3%)
Acute renal failure	2 (4.4%)
Pneumonia	2 (4.4%)
Seizure	2 (4.4%)
Cardiac arrest	1 (2.2%)
Splenic infarction	1 (2.2%)
Meningitis	1 (2.2%)
Acute respiratory distress syndrome	1 (2.2%)
Disseminated intravascular coagulation	1 (2.2%)
Presence of biliary leak	4 (8.9%)
Mortality	1 (2.2%)
	Mean ± SD
PRBCs	5.91 ± 4.48
FFP	6.36 ± 3.67
Platelet	5.89 ± 3.79
	Median (min–max)
ALT	229 units/L (21–1277)
AST	198.5 units/L (19–1000)
Re-bleed rate	1 (1–5)
GCS score	15 (3–15)
Length of hospital stay (days)	11 (1–316)
ICU stay (days)	4 (1–54)
Ward stay (days)	7 (1–262)

Abbreviations: *n*, numbers; PTD, percutaneous transhepatic biliary drainage; E-FAST, Extended Focused Assessment with Sonography in Trauma; SD, standard deviation; PRBCs, packed red blood cells; FFP, fresh frozen plasma; Min, minimum; Max, maximum; ALT, alanine aminotransferase; AST, aspartate aminotransferase; GCS, Glasgow coma scale; ICU, intensive care unit.

**Table 4:** Main characteristics of operative and nonoperative groups (*n* = 45).

Factor	Type of Treatment	
	Conservative *n* (%) (*n* = 37)	Laparotomy/ Conservative to Laparotomy *n* (%) (*n* = 8)
Age in years (mean ± SD)	27.6 ± 14.4	37.2 ± 18.6
Gender		
Male	29 (78.4%)	6 (75%)
Female	8 (21.6%)	2 (25%)
Mechanism of injury		
Blunt trauma	35 (94.6%)	4 (50%)
Non-blunt trauma	2 (5.4%)	4 (50%)
Type of injury ^†^		
Laceration	36 (97.3%)	8 (100%)
Contusion/hematoma	21 (56.8%)	2 (25%)
Hemoperitoneum	9 (5.4%)	4 (50%)
Grade		
Grade 1	4 (10.8%)	1 (12.5%)
Grade 2	10 (27%)	3 (37.5%)
Grade 3	15 (40.5%)	2 (25%)
Grade 4	7 (18.9%)	2 (25%)
Grade 5	1 (2.7%)	0
Complications	7 (18.9%)	5 (62.5%)
Positive E-FAST *	10 (28.6%)	6 (85.7%)
Hemodynamic stability		
Unstable	10 (27%)	4 (50%)
Stable	27 (73%)	4 (50%)
	Mean ± SD	Mean ± SD
PRBCs	5.59 ± 4.24	6.83 ± 5.42
FFP	6.57 ± 3.05	6.00 ± 5.09
Platelet	5.33 ± 4.08	7.00 ± 3.61
	Median (min–max)	Median (min–max)
ALT	241 (21–1277)	207.5 (63–935)
AST	198.5 (19–1000)	232.5 (39–950)
Re-bleed rate	1 (1–2)	2 (1–5)
GCS score	11.5 (5–15)	15 (5–15)
Length of hospital stay (days)	22 (8–92)	30 (15–316)
ICU stay (days)	11 (2–20)	16 (4–54)
Ward stays (days)	13.5 (4–76)	14 (4–262)

Abbreviations: *n*, numbers; SD, standard deviation; E-FAST, Extended Focused Assessment with Sonography in Trauma; PRBCs, packed red blood cells; FFP, fresh frozen plasma; Min, minimum; Max, maximum; ALT, alanine aminotransferase; AST, aspartate aminotransferase; GCS, Glasgow coma scale; ICU, intensive care unit. ^†^ Some patients had multiple types of injuries. * Three patients who had not undergone E-FAST (Extended Focused Assessment with Sonography in Trauma) were excluded from the analysis.

**Table 5:** Descriptive analysis of variables linked to hemodynamic status (*n* = 45) *.

Factor	Hemodynamic Stability	
	Unstable *n* (%) (*n* = 14)	Stable *n* (%) (*n* = 31)
Age in years (mean ± SD)	28.9 ± 14.0	29.5 ± 16.3
Gender		
Male	12 (85.7%)	23 (74.2%)
Female	2 (14.3%)	8 (25.8%)
Mechanism of injury		
Blunt trauma	12 (85.7%)	27 (87.1%)
Non-blunt trauma	2 (14.3%)	4 (12.9%)
Type of injury ^†^		
Laceration	14 (100%)	30 (96.8%)
Contusion/hematoma	5 (35.7%)	18 (58.1%)
Hemoperitoneum	5 (35.7%)	8 (25.8%)
Grade		
Grade 1	1 (7.1%)	4 (12.9%)
Grade 2	4 (28.6%)	9 (29%)
Grade 3	7 (50%)	10 (32.3%)
Grade 4	2 (14.3%)	7 (22.6%)
Grade 5	0	1 (3.2%)
Complications	8 (57.1%)	4 (12.9%)
Positive E-FAST *	5 (38.5%)	11 (37.9%)
	Mean ± SD	Mean ± SD
PRBCs	8.36 ± 4.5	3.67 ± 3.2
FFP	7.29 ± 4.31	4.75 ± 1.5
Platelet	7 ± 4	3.67 ± 2.52
	Median (min–max)	Median (min–max)
ALT	165.5 (34–935)	259 (21–1277)
AST	155.5 (27–950)	230.5 (19–1000)
Re-bleed rate	1 (1–5)	1 (1–3)
GCS score	10 (5–15)	15 (5–15)
Length of hospital stay (days)	29 (8–316)	19 (10–30)
ICU stay (days)	16 (2–54)	9 (4–16)
Ward stays (days)	19 (4–262)	9 (4–18)

Abbreviations: *n*, numbers; SD, standard deviation; E-FAST, Extended Focused Assessment with Sonography in Trauma; PRBCs, packed red blood cells; FFP, fresh frozen plasma; Min, minimum; Max, maximum; ALT, alanine aminotransferase; AST, aspartate aminotransferase; GCS, Glasgow coma scale; ICU, intensive care unit. ^†^ Some patients had multiple types of injuries. * Three patients who had not undergone E-FAST (Extended Focused Assessment with Sonography in Trauma) were excluded from the analysis.

## Data Availability

The data that support the findings of this study are available from the corresponding author upon reasonable request.
